# Breeding pattern of *Oreochromis niloticus* (Linnaeus, 1758) versus native congeneric species, *Oreochromis macrochir* (Boulinger, 1912), in the upper Kabompo River, northwest of Zambia

**DOI:** 10.1002/ece3.8377

**Published:** 2021-11-18

**Authors:** Arthertone Jere, Wilson W. L. Jere, Austin Mtethiwa, Daud Kassam

**Affiliations:** ^1^ Department of Aquaculture and Fisheries Science Africa Centre of Excellence in Aquaculture and Fisheries (AquaFish ACE) Faculty of Natural Resources Lilongwe University of Agriculture and Natural Resources Lilongwe Malawi; ^2^ Department of Fisheries, Extension Services Ministry of Fisheries and Livestock Solwezi Zambia

**Keywords:** exotic fishes, invasive patterns, Kabompo River, outbreeding, spawning events, Zambia

## Abstract

Investigating the determinants of the reproductive biology of fishes is an essential component of fisheries research. Tilapia breeding patterns were investigated to determine the impact of non‐native *Oreochromis niloticus* on the native congeneric *Oreochromis macrochir* in the upper Kabompo River in the Northwest of Zambia using the gonadosomatic index and the sex ratios. *Oreochromis niloticus* was the most abundant fish caught (221, 63.5%) than *O*. *macrochir* (127, 36.5%). Results showed that the overall gonadosomatic index means of *O*. *macrochir* in both sections were similar. *Oreochromis macrochir* bred in December and February–March, with no reproduction in June. However, *O*. *niloticus* in the invaded section indicated all year reproduction through reduced spawning in May–June, with increased spawning activity in February–March. The sex ratio (females: males) was 1:1.3 and 1:1.7 for *O*. *niloticus* and *O*. *macrochir*, respectively, and both significantly deviated from the sex ratio of 1:1 (ꭓ^2^ = 8.42 and 9.37, *p* < .05). Our study has revealed that *O*. *niloticus* was able to spawn across all sampled months with a 23% higher breeding population than *O*. *macrochir*, which might explain the suppression in the abundance of native *O*. *macrochir*. Due to the superior breeding patterns of *O*. *niloticus*, fisheries, wildlife, and aquaculture practitioners need to make contingency plans to alleviate its impacts further downstream of the Kabompo River.

## Graphical abstract

Breeding pattern of *Oreochromis niloticus* (Linnaeus, 1758) (Pisces: Cichlidae) versus native congeneric species, *Oreochromis macrochir* (Boulinger, 1912), in the upper Kabompo River, northwest of Zambia, was performed to understand the impact of invasion on native species.

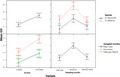

## 1 INTRODUCTION

The introduction of invasive alien species in an ecosystem poses a greater risk to cause ecological disruption in stable environments once it establishes (Ellender et al., 2018; Jere et al., 2021; Lowe et al., 2018; Vicente et al., 2011). This has resulted in possible harmful relationships between invasive alien species and native species (Le Roux et al., 2020; Zengeya et al., 2015). Anthropogenic activities are the leading cause of the intentional and unintentional introduction of invasive alien species over the world (Fonseca–Alves et al., 2011). Tilapias (Cichlidae) are among the African native species that have been introduced to other continents around the world, because of their ability to reproduce frequently and attainment of early sexual maturing (Azua et al., 2017; van Wilgen et al., 2020). On the contrary, tilapias can have negative effects on ecosystems, causing serious modification on the aquatic community dynamics and further reducing biodiversity (Gozlan et al., 2010; Groombridge, 1992; Jere et al., 2021; Le Roux et al., 2020). Generally, the introduction of invasive alien species threatens the stability of aquatic environments, triggering competition and thereafter suppressing the native communities leading to the extinction of the native species (Gozlan et al., 2010; Lowe et al., 2018; Zengeya et al., 2020).

In sub‐Saharan Africa, most freshwaters have been invaded by alien fish species (Zengeya et al., 2015). The characteristics of invaders include early maturity and a fast growth rate that makes them successful and impact negatively the diversity of native species (Beyruth et al., 2004; Espinola et al., 2010; Jere et al., 2021). In the genus *Oreochromis*, males build nests for spawning and develop secondary sexual structures (Beyruth et al., 2004; Gozlan et al., 2010; Lowe et al., 2018; Turner & Robinson, 2000). These attributes make invasive alien species to be more dominant in areas outside their normal range than native species and may create an ecosystem population imbalance. This population imbalance of native species may cause the native ecosystem to be more vulnerable to resist further impacts of invasion (Jere et al., 2021). Moreover, invasive alien species are difficult to control, in many instances where chemical or physical means were employed; no significant eradication has been achieved (Groombridge, 1992; Jere et al., 2021). This could suggest that a strict policy on the introduction of alien species is the best means to avoid control invasion.

Nile tilapia, *Oreochromis niloticus* (Linnaeus, 1758), is among the most preferred fish species for aquaculture purposes, despite its ability to reduce local biodiversity through competition with native species (Zengeya et al., 2020). However, some countries have introduced *O*. *niloticus* to increase local fish diversity and supply food fish to local people (Azua et al., 2017). The species is preferred for aquaculture because of its ability to tolerate a wide range of environmental conditions (Grammer et al., 2012; van Wilgen et al., 2020). In Zambia, *O*. *niloticus* was introduced in the late 1980s by the Department of Fisheries for research and development in aquaculture (Bbole et al., 2014; DoF, 2018; FAO, 2012; Kenzo & Mazingaliwa, 2002).

To understand the impacts of invasive alien species, breeding patterns is one way in which these impacts can be comprehended. To achieve this, gonad staging using gonadosomatic index (GSI) is the accurate and reliable means to determine the reproductive biology of fish (Rodi & Mackie, 2001). This method is an important tool for elucidating breeding differences between congeneric fish species (Rodi & Mackie, 2001; Gozlan et al., 2010; van Wilgen et al., 2020). Therefore, results provided by the gonadosomatic index method can be used to determine the spawning events of species. This is an important predictor of the impacts of invasive alien species in the aquatic ecosystem (Lowe et al., 2018; van Wilgen et al., 2020).

The impacts of invasion by exotic species vary spatially and temporarily and tend to be context‐dependent in the subtropical lotic systems (Rouget et al., 2002). One way in which exotic species impact indigenous species is through rapid spawning outside its normal range, which may lead to dominance and suppression of native species. It is known that there is a viable population of exotic *O*. *niloticus* present in the upper Kabompo River, but its reproductive biology impacts on the native species have not been reported (Bok & Bills, 2012; DoF, 2018). In this study, we investigated the breeding patterns of native *O*. *macrochir* and exotic *O*. *niloticus* to better understand reproductive activity interaction (Lowe et al., 2018). This was to help explain the abundance of *O*. *niloticus* than the native congeneric species in the upper Kabompo River. The main hypothesis tested was that exotic *O*. *niloticus* would show similar breeding patterns with native congeneric *O*. *macrochir* in the upper Kabompo. This hypothesis was tested using the gonadosomatic index (GSI) to understand the spawning biology and sex ratio to investigate the reproductive potential for both species. This method used here gives us clues on the spawning biology of species and a good understanding of the reproductive potential of the species. The study will help to clarify the increased dominance of *O*. *niloticus* over the native *O*. *macrochir* and assist in developing a fisheries management plan aimed at reducing the impacts of fish invasion.

## 2 MATERIALS AND METHODS

### 2.1 Description of the study area

**FIGURE 1:**
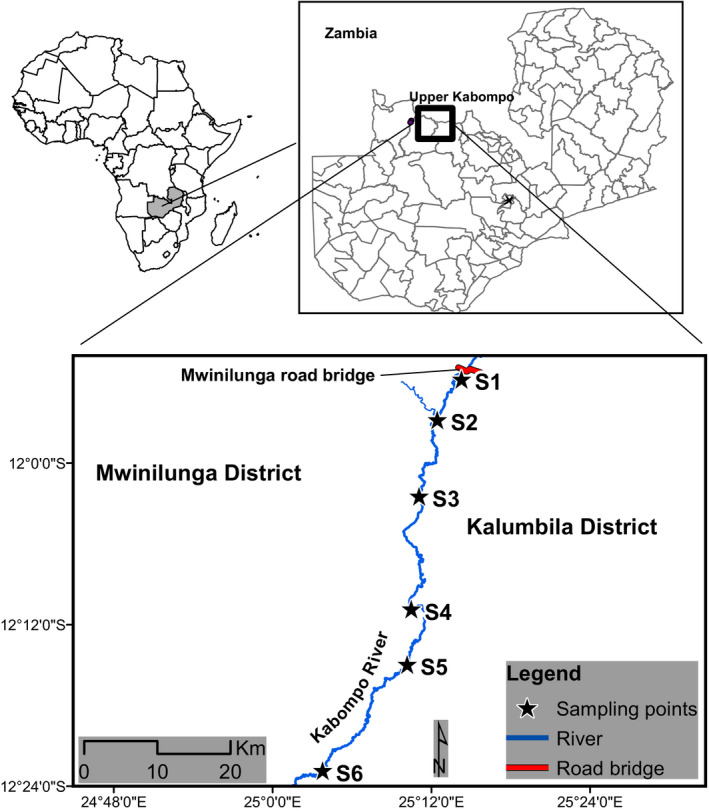
Map of the study area—upper Kabompo River

The study was conducted in the upper part of the Kabompo River, and its source is in the North Western Province of Zambia (Figure 1). The province is endowed with numerous supplies of inland flowing waters that are suitable for fish farming and fisheries. The study area stretches approximately 45 km in length from the source of the Kabompo River (latitude 25.2414 to 25.044156 E and longitude 11.8973 to −12.369120 S, respectively). The river has a natural waterfall that acts as a barrier drops‐off in the river gorge at approximately 40‐ to 50‐m elevation and disappears at 1.5km before surfacing (AES, 2014; DoF, 2018; Jere et al., 2021). Upper Kabompo is part of the Kabompo River and is one of the major tributaries of the Zambezi River. It originates at an altitude of approximately 1500 m above mean sea level (amsl) in the highlands, which forms the watershed between the Zambezi and Congo rivers. It has a water surface area of about 1473 km^2^, with abundant fish landing sites. The study site is one of the sources of brood fish for the Aquaculture Breeding Programme in Zambia (DoF, 2018). It was also chosen because it is the most active fishing area and of economic importance to the surrounding villages as a source of livelihood (Bok & Bills, 2012).

### 2.2 Sampling procedure

Six sampling points were chosen at 4.5‐km intervals, covering approximately 30 km of the river stretch (Figure 1). The study area was divided into two sections: the uninvaded section with three sampling points that was not invaded by *O*. *niloticus* following the aquatic biodiversity study report by Bok and Bills (2012) and AES (2014); and the invaded section with three sampling points that was invaded by *O*. *niloticus* and is located downstream after the natural waterfall (AES, 2014; Bok & Bills, 2012; DoF, 2018). At each sampling point, a 200‐m stretch was covered involving five available microhabitat types (runs, riffles, vegetative thicket point, open poor [near fisheries landing area], and the tributaries of the river). Sampling was conducted for three main sampled months of the year comprised of December 2019 (average temperature of 28.12°C ± 0.18), January–February 2020 (average temperature of 24.08°C ± 0.19), and May–June 2020 (average temperature of 17.01°C ± 0.17). In each period, sampling was conducted daily for 4 weeks in a month, out of which two weeks were allocated in the sampling section per month. The fish were caught in the early mornings (06:00 h) and evenings (17:00 h), because in these hours, all the fish were fresh and easy to identify before spoilage takes place.

One sampling was assumed to be invaded, while the other section was uninvaded following the aquatic biodiversity report (AES, 2014; DoF, 2018). This provided a platform for comparison of the two species under review. Invasive alien *O*. *niloticus* was compared with native congeneric *O*. *macrochir* to reveal their breeding patterns and help to understand the impact invasive alien species have on the native species. The fish fauna in most Zambian rivers is dominated by *Oreochromis mortimeri*, *Tilapia sparmani*, *Oreochomis andersonii*, *Coptodon rendalli*, *Oreochromis tanganikae*, *Oreochromis macrochir*, *Oreochomis niloticus*, and other miscellaneous fishes (DoF, 2018; Kenzo & Mazingaliwa, 2002). The comparison between *O*. *niloticus* and native *O*. *macrochir* was based on the fact that it was the only species found in both juvenile and adult stages than other natives and the most abundant native congeneric species found in both sections of the river during the survey. The first comparison focused on the breeding patterns of *O*. *niloticus* and *O*. *macrochir* in sampling points of the invaded section. The second comparison was meant to investigate any differences in the breeding pattern between the *O*. *macrochir* present in invaded and uninvaded sections; to clarify whether invasion could trigger changes.

### 2.3 Fish sampling

Fish were collected separately from the runs, riffles, vegetative thicket points, and open pools using different fishing gears. The following fishing gears were used in the fish collection: gill nets ranging in mesh size from 2 to 6.5 cm with 3‐m depth and 30‐m length; two double‐ended fyke nets made from 20‐mm stretched multifilament netting with 75‐cm D‐ends separated by an 8‐meter leader; an LR‐24 Electrofisher (Smith‐Root) with 400‐watt electrical output; and a beach seine net of 25‐meter length and 3‐m depth. The fyke and gill nets were deployed parallel to the bank in slow‐flowing water where boat access was not restricted by fish weirs or fallen trees and inspected in the morning (06:00 h) and afternoon (17:00 h) for the sampling period. Electric fishing and seining were operated during the daytime and over three days per week within each sampling point.

The caught fish species were sorted and identified where possible using the reference material by Kenzo and Mazingaliwa (2002). A total of 1548 fish were collected, of which 921 were *O*. *niloticus* and 627 were native *O*. *macrochir*. The collected specimens were immediately sexed, after which they were stored in ice and taken to the laboratory for further examination. For the specimens that we were able to dissect in the field, their gonads were weighed to the nearest 0.01 gm, fixed in 10% formalin, and taken to the laboratory for microscopic examination.

The preserved gonad samples from the field were removed from the formalin solution in the laboratory for GSI determination. Microscopic staging using SHT was also used in the processing of the samples under a light microscope for examination of sex and gonad development from gametes. This process is important as it supplements the GSI method in determining the reproductive timing of the fish (Hilge, 1977). To do this, the whole specimens were blotted dry and reweighed to the nearest 0.01gm. Individual gonad transverse portion was removed and then processed using standard histological techniques (SHT) to give 5‐ to 8‐μm sections that were stained for microscopic examination using the procedure by Harris's hematoxylin and eosin (H&E) (Hilge, 1977). The removal of the transverse portion of gonads from the middle region of one lobe was done to determine whether uniformity exists in the development of gametes. Only gonads of 689 examined specimens that showed mature males and females represented in all reproductive stages (1–6, females and 1–5, males) were considered. These measurements were collated in a developed Microsoft Excel spreadsheet for data entry for computation of GSI and sex ratio. Gonadosomatic index (GSI) of the female and male for each species of the collected samples was determined separately using the formula.

$$\text{GSI}=\frac{\text{GW}}{\text{EBW}}\times 100$$

where GW = gonad weight (grams), EBW = eviscerated body weight (grams).

The gonad maturity stages (immature/virgin, early maturing, developing, pre‐spawning, spawning, and spent) were visually investigated following the Nagelkerke and Sibbing (1996).

### 2.4 Statistical analyses

Data were entered into Microsoft Excel 2007 (summation, average, and percentages) for computation of GIS and sex proportion of *O*. *niloticus* and *O*. *macrochir* in the upper Kabompo River. The GSI mean data satisfied the parametric assumptions after subjecting them to the normality and homoskedastic tests (Sokal & Rohlf, 1995). Thereafter, two‐way ANOVA using interaction contrast was then used to test the differences in the means of a gonadosomatic index of both males and females of *O*. *niloticus* and *O*. *macrochir* (Sokal & Rohlf, 1995). Equally, a chi‐squared test was performed to test for whether each species mean sex ratio differed from the expected sex ratio of 1:1 (Kings, 1995). All statistical tests were performed using the R software package (R Core Team, 2019).

## 3 RESULTS

The results have revealed similar maturity stages for both sexes of *O*. *niloticus* and *O*. *macrochir* as illustrated by the gonad development stages in Tables 1 and 2. The percentage of the gonad development stage of the fish caught in each sampling period were categorized into six development stages for females and five development stages for males. Developmental stages of gonads for both *O*. *niloticus* and *O*. *macrochir* showed that immature/virgin, early maturing, developing, pre‐spawning, spawning, and spent stages were found in different size classes in different catches. Gonads of both male and female fishes were examined from three different months. It was found that females of both *O*. *niloticus* and *O*. *macrochir* represented all reproductive stages (1–6) and different gonadosomatic indices (GSI: 0.2 to 5.5%). For males, only reproductive stages (1–5) were represented with gonadosomatic index (GSI) of 0.2 to 5.8% for *O*. *niloticus* and GSI of 0.2% to 6% for *O*. *macrochir*. In general, the highest GSI for both species were recorded in January–February 2020 and thereafter in December. Accordingly, the females’ gonad maturation stages of *O*. *niloticus* were varying, indicating 13% of the fish analyzed were in immature, 20% in early maturing, 20% in developing, 10% in pre‐spawning, 20% in spawning, and 17% in spent stages. Its average ovum diameter ranged from 0.0615 to 0.9854 mm (Table 1). The females’ gonad maturation stages of *O*. *macrochir* were varying, indicating 14% of the fish analyzed were in immature, 11% in early maturing, 7% in developing, 24% in pre‐spawning, 27% in spawning, and 17% in spent stages. Its average ovum diameter ranged from 0.0242 to 0.8304 mm (Table 1). The males’ gonad maturation stages of *O*. *niloticus* were varying, indicating that 10%, 17%, 25%, 18%, and 30% of the fish analyzed were in immature/virgin, immature developing, mature resting, developed, and spawning stages, respectively. Its average testis diameter ranged from 934 to 4674 mm (Table 2). The gonad maturation stages for males of *O*. *macrochir were* varying, indicating that 15%, 12%, 18%, 41%, and 14% of the fish analyzed were in immature/virgin, immature developing, mature resting, developed, and spawning stages, respectively. Its average testis diameter ranged from 845 to 5696 mm (Table 2).

**TABLE 1:** Microscopic stages from histological analysis for the females of *Oreochromis macrochir* (*n *= 150) and *Oreochromis niloticus* (*n *= 238) specimens were analyzed in the upper Kabompo River

Stage	Degree of maturity	*O*. *macrochir*		*O*. *niloticus*		Description
		Percentage of fish analyzed	Average ovum diameter (mm)	Percentage of fish analyzed	Average ovum diameter (mm)	
1	Immature and resting adult	14	0.0425	13	0.334	Very small ovaries and thin thread‐like pale in color
2	Early maturing	11	0.0344	20	0.0712	Slightly larger ovaries and increase in weight and with minute opaque whitish eggs occupying half of the body cavity
3	Developing	7	0.0838	20	0.0615	2/3 of the abdominal cavity is occupied with ovaries with pale yellow eggs
4	Pre‐spawning	24	0.8304	10	0.8451	Deep yellow ripe ova, almost entire body cavity occupied with ovaries, with a large number of big, turgid, spherical, and translucent ova
5	Spawning	27	0.5187	20	0.9854	Transparent ovary walls, ripped eggs are visible through the walls of the ovary and some ripped eggs in the oviduct
6	Spent	17	0.0242	17	0.0892	Ovaries are flaccid shrink, sac‐like, and reduced in size. Ripped unspawned darkened eggs and with a large number of small ova

**TABLE 2:** Microscopic stages from histological analysis for the males of *Oreochromis macrochir* (*n *= 130) and *Oreochromis niloticus* (*n *= 171) specimens were analyzed in the upper Kabompo River

Stage	Degree of maturity	*O*. *macrochir*		*O*. *niloticus*		Description
		Percentage of fish analyzed	Average testis diameter (mm)	Percentage of fish analyzed	Average testis diameter (mm)	
1	Immature/Virgin	15	845	10	934	Small testis with a smooth appearance and opaque, ivory color. Difficult to distinguish the testis stage from juveniles
2	Immature developing	12	1245	17	1169	Testes not only are small with features of the "virgin" testes but also produce milt when squeezed
3	Mature resting	18	2456	25	3125	Opaque and small testis and strap‐like. Very little or no milt is extruded from the transverse section when squeezed
4	Developed	41	5696	18	4674	Large testes and opaque with ivory‐like color. The exterior dorsal blood vessel is large, and small blood vessels are usually present. Milt can usually be squeezed out. Testes may also be misidentified as resting or virgin
5	Spawning	14	3045	30	2945	Ripe testis with larger exterior blood vessels. Milt is the release with little or no pressure on the abdomen or when the testis is cut

**FIGURE 2:**
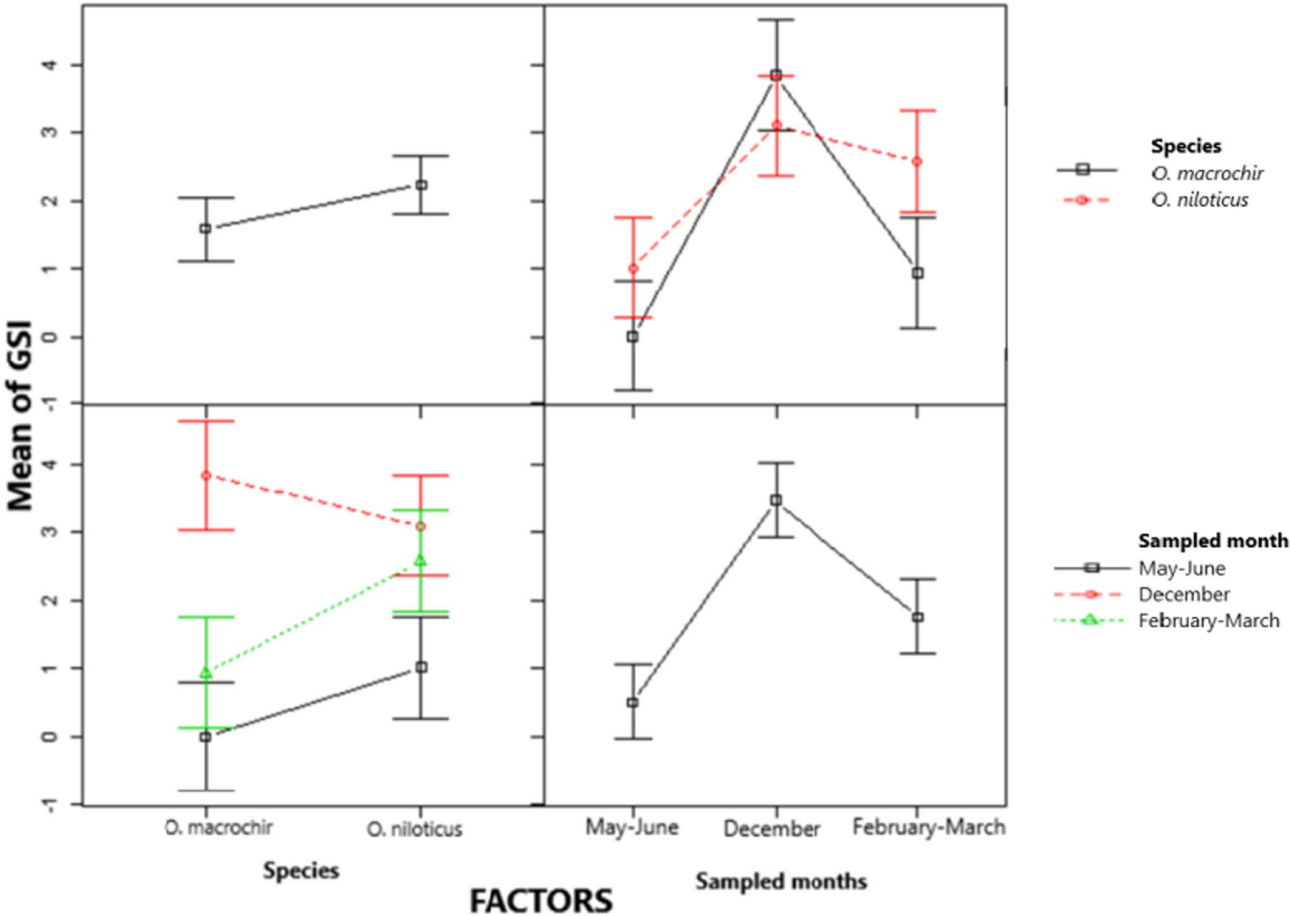
Mean gonadosomatic index variation for males of exotic *Oreochromis niloticus* and *native Oreochromis macrochir* in the upper Kabompo River

**FIGURE 3:**
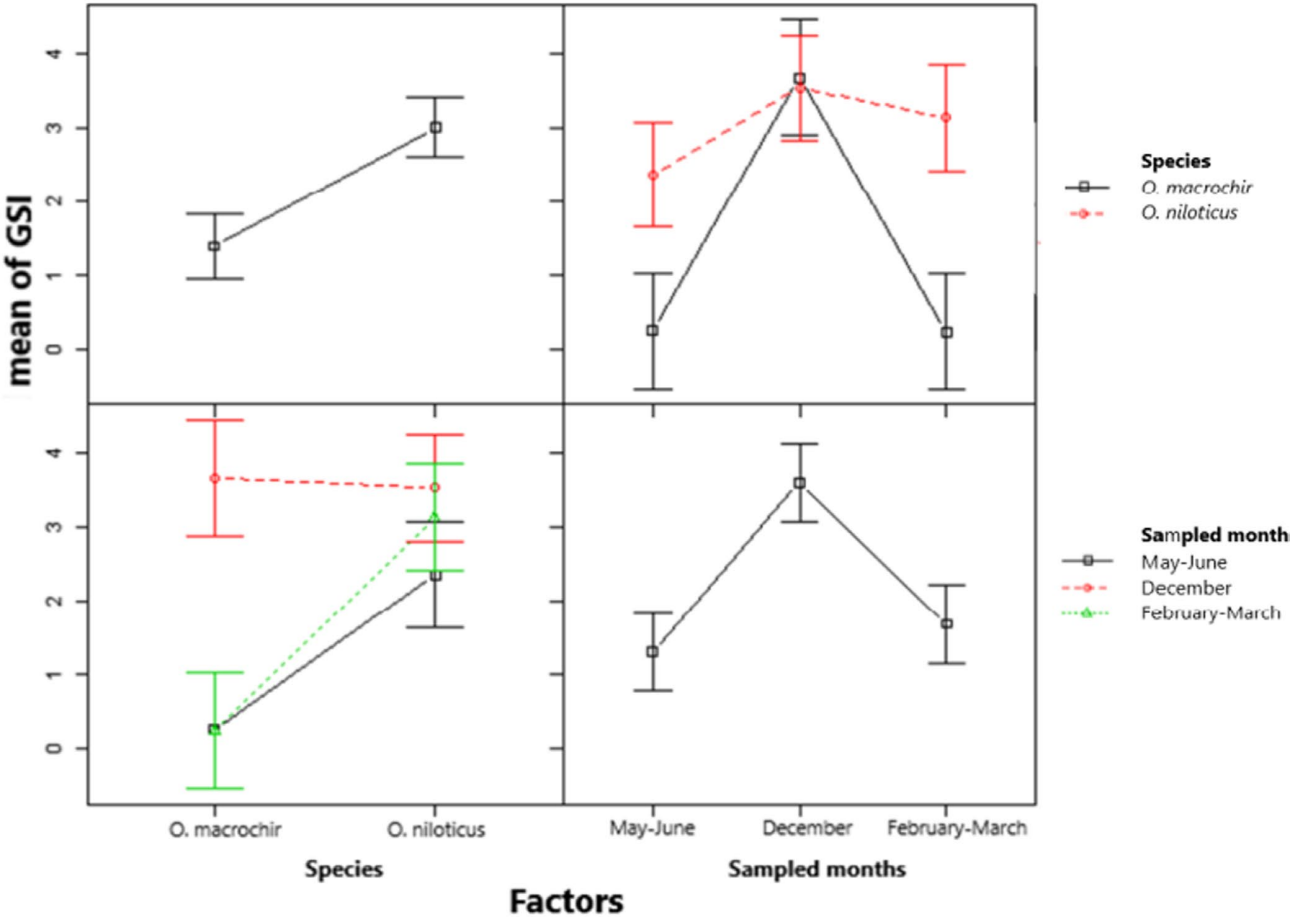
Mean gonadosomatic index variation for females of exotic *Oreochromis niloticus* and *native Oreochromis macrochir* in the upper Kabompo River

**FIGURE 4:**
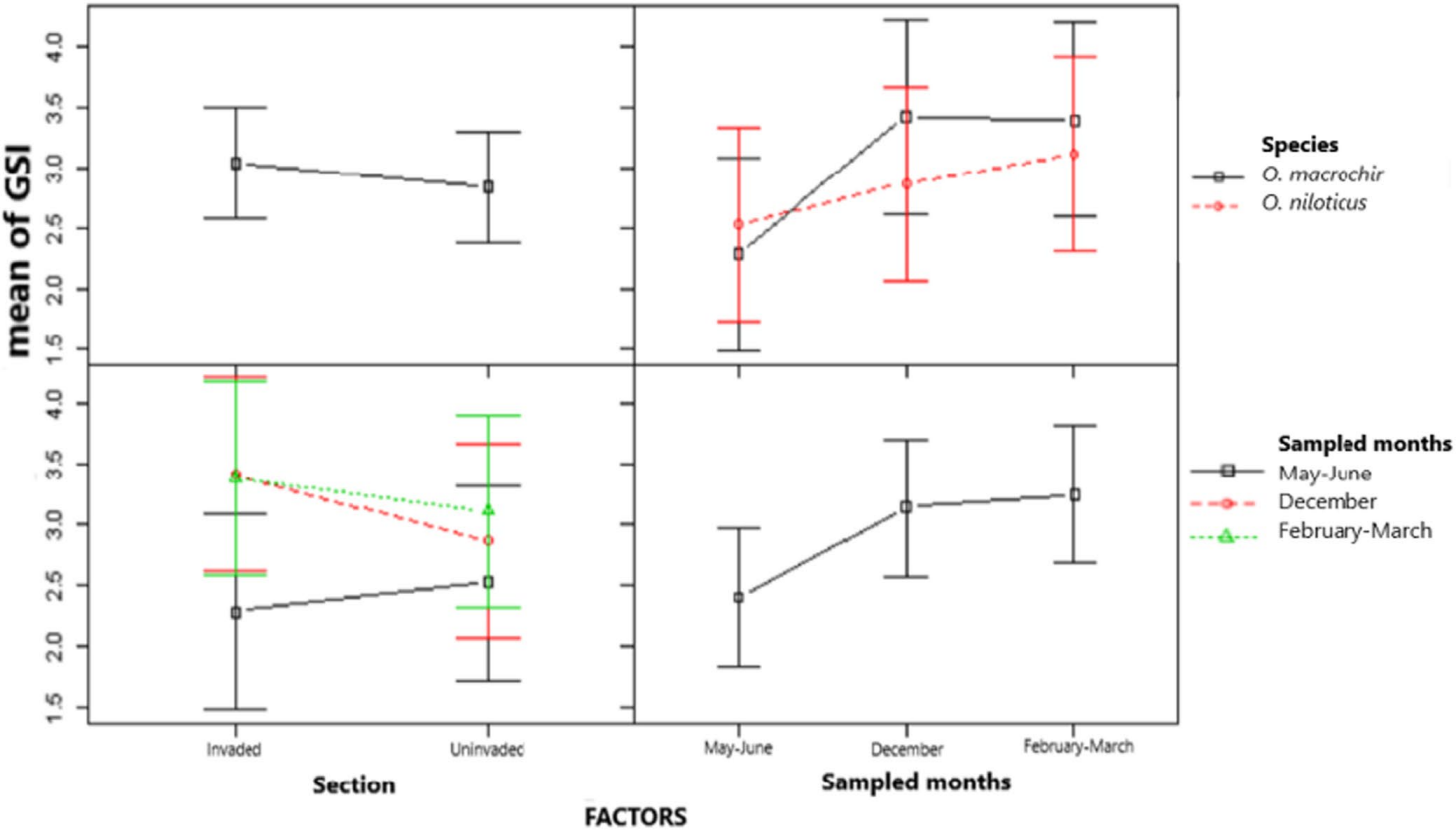
Mean gonadosomatic index variation for females of *native Oreochromis macrochir* in invaded and uninvaded sections of the upper Kabompo River

**FIGURE 5:**
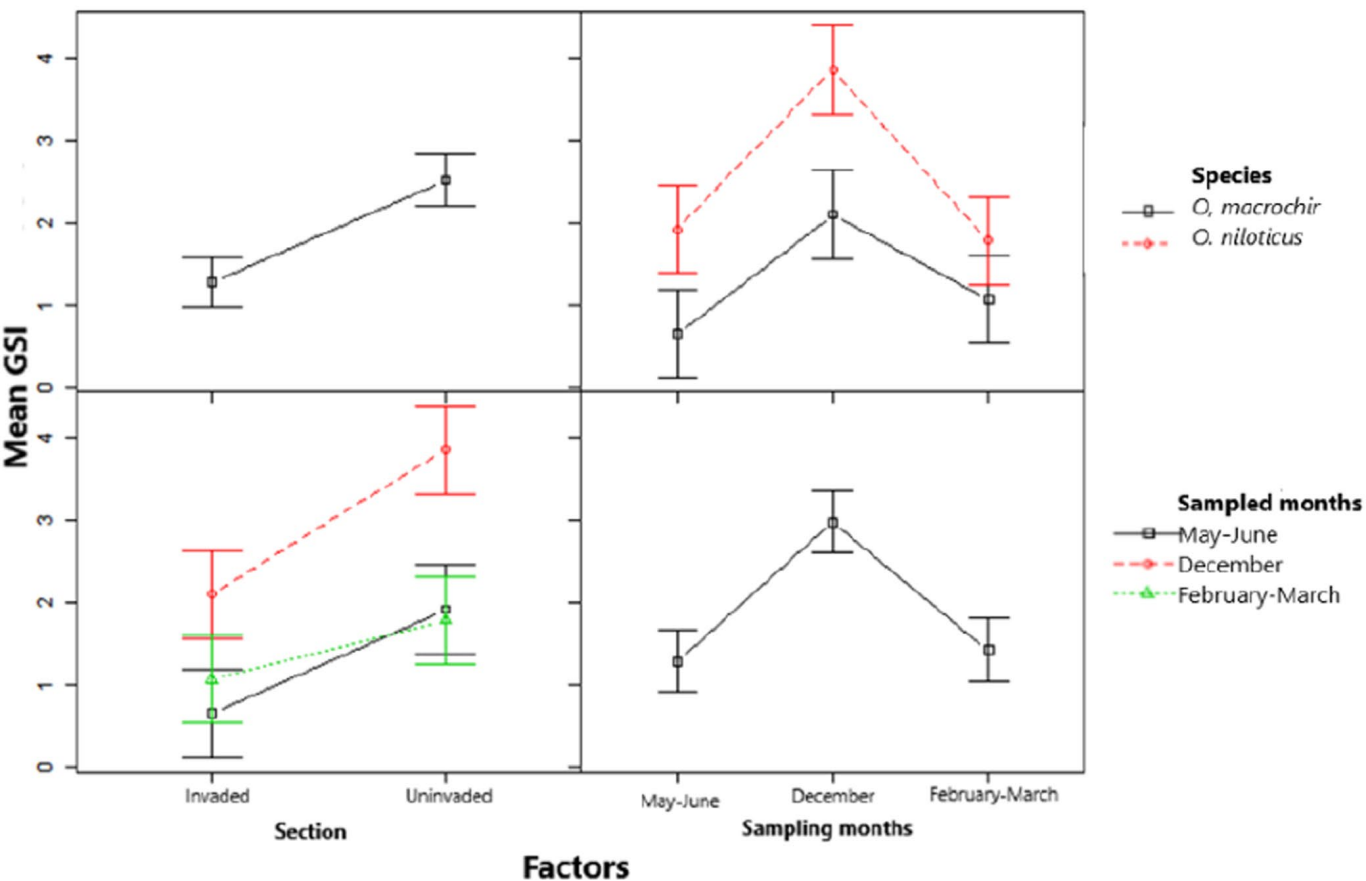
Mean gonadosomatic index variation for males of *native Oreochromis macrochir* in invaded and uninvaded sections of the upper Kabompo River

The mean variation of GSI for males and females of *O*. *niloticus* and *O*. *macrochir* for all the three sampling periods was significantly different (*F*(3, 32) = 1.045, *p* = .0628 and *F*(3, 47) = 1.409, *p* = .0041) (Figures 2 and 3, respectively). In contrast to the mean variation of GSI, the mean GSI was not significantly different (*F*(3, 39) = 1.343, *p* = .6217 and *F*(3, 41) = 1.335, *p* = .8144) in males and females of native *O*. *macrochir* in both invaded and uninvaded section of the river in all the three sampled months (Figures 4 and 5, respectively). The study also showed a consistent pattern of spawning in *O*. *niloticus* in three sampled months as indicated during December 2019, January–March 2020, and May–June 2020 survey period. In the case of *O*. *macrochir* was found naturally spawning more during January–March 2020 than December, while in May–June, spawning had completely stopped and only the fish in the immature/virgin stage were collected from the sampling sites.

**FIGURE 6:**
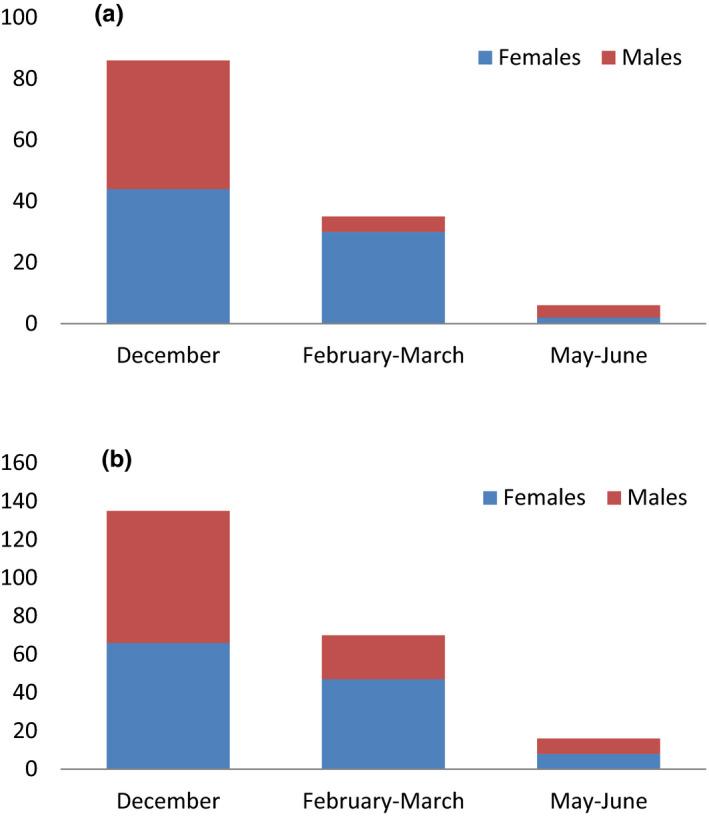
Abundance of males and females for (a) *Oreochromis niloticus* and (b) *Oreochromis macrochir* for the sampled months in the upper Kabompo River

The study has also revealed that the native *O*. *macrochir* was found to have a sex ratio of 76 females (59.8%) and 51 males (40.2%), while *O*. *niloticus* had a sex ratio of 121 females (54.8%) and 100 males (45.3%) (Figure 6). Females were more numerous in all the 3 sampled months for both species (Figure 2). Sex ratio was 1:1.34 (females: males) and significantly deviated from the sex ratio 1:1 (ꭓ^2^(2, 388) = 8.42, *p* = .0117) for *O*. *niloticus*, while for *O*. *macrochir*, sex ratio was 1:1.72 (females: males) and significantly deviated from the sex ratio 1:1 (ꭓ^2^(2, 301) = 9.37, *p* = .0321).

## 4 DISCUSSION

One way in which exotic species impact indigenous species is through rapid spawning outside its normal range, which may lead to dominance and suppression of native species. Therefore, our study aimed at investigating breeding patterns of *O*. *niloticus* on the native congeneric species, to enable us better understand the impacts of invasion. The study revealed that the *Oreochromis niloticus* spawned throughout three sampled periods as shown by the presence of gonads in an advanced stage of maturation than native congeneric species in the upper Kabompo River. Our hypothesis that indicated that *O*. *niloticus* and *O*. *macrochir* have similar breeding patterns was rejected, because of the observed difference in the GSI means in all the sampled months of the year. The different size classes of *O*. *niloticus* catch composition observed in the study implied that it has established a breeding population in parts of the lower stretch of the upper Kabompo River. This stretch of the river constitutes the bulk of the cichlids catches by local fishers, and fish abundances between *O*. *niloticus* and *O*. *macrochir* were different. Fish catches of *O*. *niloticus* were more abundant than those of *O*. *macrochir*, with the presence of all reproductive stages (1–6) for females and (1–5) for males. The findings from a similar study on the Kafue River, the southern region of Zambia, were similar (Bbole et al., 2014; Marshall, 2011). Similarly, Lake Kariba that receives water inflow from the Zambezi River showed high catches of *O*. *niloticus* than the native congeneric species (Marshall, 2011; Zengeya et al., 2020).

Despite the variation in the gonad stage of males and females for *O*. *niloticus*, results showed continuous spawning in the sampled months (December 2019, January to February 2020, and May to June 2020), in the upper Kabompo River. Similarly, previous studies have shown that reproductive activity of *O*. *niloticus* has been reported to be continuous (sampled period) in females (De Silva & Chandrasoma, 1980; Zengeya et al., 2015). Stewart (1988) and Gomez‐Marquez (1998) also found that *O*. *niloticus* females breed more than once in a sampled period. Our study provided the first evidence of the May–June spawning of *O*. *niloticus* in the upper Kabompo River forming feral populations as different size classes of cohorts. However, *O*. *macrochir* was found to spawn significantly higher during December than the February–March months, and in the period of May–June, it stopped to spawn completely. This could suggest that the environmental conditions were not favorable compared with other sampled months. A similar study indicated that environmental factors such as low water temperature play a critical role in influencing the frequency of spawning for most tilapia. This could be the reason to explain why *O*. *macrochir* was found spawning only in two sampled months of the year. Similarly, in Mexico tilapia, frequent spawning events were observed in the year when water temperatures were favorable (Gomez‐Marquez, 1998; Le Maitre et al., 2020; Lowe et al., 2018).

*Oreochromis niloticus* is associated with environmental tolerance, early sexual maturity, and rapid colonization (Ellender et al., 2018; Pérez et al., 2006; Russell et al., 2012). The aforementioned characteristics are considered critical in facilitating a successful invasion and establishment of this fish in the river, which may ultimately affect the dominance of the native congeneric species (*O*. *macrochir*). In this study, the observed GSI values observed in all the sampled periods are consistent with year‐round spawning and are similar to the GSI values reported by Lowe et al. (2018). In *O*. *macrochir*, only two sampled months of the year were observed in our study and suggested that it was not able to spawn throughout the year. The observed GSI values coupled with the occurrence of mature females and males in the three sampled months, and recruitment of young fish into larger size classes strongly suggests that *O*. *niloticus* is well established in the upper section of the Kabompo. *Oreochromis macrochir* is an important species and one of the most favored and recommended native fish species for aquaculture in Zambia. Many native congeneric species including *O*. *macrochir* are critical species for the Aquaculture Breeding Programme in Zambia. The observed difference in the spawning events may negatively affect the catches, as most catches will be dominated by *O*. *niloticus*. This eventually could trigger hybridization between them, as documented by Bbole et al. (2014) in the Kafue River. This may lead to the extinction of native congeneric species and also may affect the purity of the broodstock meant for the improvement of the native breeding population for the aquaculture industry.

The sex ratio observed from our study indicated that *O*. *macrochir* and *O*. *niloticus* were different in numbers, and it was deviated from the expected 1:1 (male: female) and was not significant. These findings were supported by other studies on the *Oreochromis* species in different aquatic environments (Zengeya et al., 2020). On the one hand, Lowe et al. (2018) did indicate that the sex ratio for *O*. *aureus* was 2.6: 1 (male: female) and was taken to be normal. On the other hand, Wilson et al. (2020) mentioned that in cichlids for every male, there is a female individual and this supported our study results. Similarly, Fryer and Iles (1972) also cited that the sex ratio varies significantly from species to species and across months of the year; however, in the majority of cases it is relatively close to 1 in the same population. The sex ratio variation indicated in different studies points to the fact that fertilization of the eggs may have been concluded and males possibly emigrated from the spawning sites to the feeding areas leaving females behind incubating the young (Zengeya et al., 2020). The sex ratio helps to shape and maintain an ecological species population balance and may help to resist invasion by invasive alien species (Zengeya et al., 2020). The sex ratio of *O*. *niloticus* was similar to that of native congeneric species and could not help to explain the difference in the abundance between the two species in the upper Kabompo River.

Collectively, our results indicate that *O*. *niloticus* invasion has increasingly dominated most sampling points of the invaded section in the upper Kabompo River contributing distinctively to the fishery, which is seriously considered in native fish species diversity sustainability. *Oreochromis niloticus* impacted negatively on the native congeneric species abundance and distribution. This was evident as *O*. *niloticus* was found breeding throughout the sampled months than *O*. *macrochir* that only bred in two sampled months of the year. However, native *O*. *macrochir* did not show any significant differences in reproductive activity in all the sampled months of the year. Our study did confirm that invasion by *O*. *niloticus* was different from native *O*. *macrochir*, but could not show that its reproductive activity was affected in the upper Kabompo River.

We recommended that further studies understand the possibility of ecosystem‐wide consequences of invasion and determine the extent to which other native fish assemblages respond throughout the continually expanding range of *O*. *niloticus* in the upper Kabompo River.

## AUTHOR CONTRIBUTIONS

**Arthertone Jere:** Conceptualization (equal); Data curation (equal); Formal analysis (equal); Investigation (equal); Methodology (equal); Writing‐original draft (equal); Writing‐review & editing (equal). **Wilson W. L. Jere:** Data curation (equal); Formal analysis (equal); Supervision (equal); Writing‐review & editing (equal). **Austin Mtethiwa:** Supervision (equal); Validation (equal); Visualization (equal); Writing‐review & editing (equal). **Daud Kassam:** Conceptualization (equal); Methodology (equal); Supervision (equal); Validation (equal); Visualization (equal); Writing‐review & editing (equal).

## Data Availability

The data that have been used in this study are available, and the Dryad data repository will be used to archive the data. The authors cited in this document have acknowledged that data should be stored in the Dryad data repository at https://doi.org/10.5061/dryad.sj3tx9661.
